# Anti-tumor effects of the histone deacetylase inhibitor vorinostat on canine urothelial carcinoma cells

**DOI:** 10.1371/journal.pone.0218382

**Published:** 2019-06-17

**Authors:** Shotaro Eto, Kohei Saeki, Ryohei Yoshitake, Sho Yoshimoto, Masahiro Shinada, Namiko Ikeda, Satoshi Kamoto, Yuiko Tanaka, Daiki Kato, Shingo Maeda, Masaya Tsuboi, James Chambers, Kazuyuki Uchida, Ryohei Nishimura, Takayuki Nakagawa

**Affiliations:** 1 Laboratory of Veterinary Surgery, Graduate School of Agricultural and Life Sciences, University of Tokyo, Bunkyo-ku, Tokyo, Japan; 2 Laboratory of Veterinary Clinical Pathology, Graduate School of Agricultural and Life Sciences, University of Tokyo, Bunkyo-ku, Tokyo, Japan; 3 Laboratory of Veterinary Pathology, Graduate School of Agricultural and Life Sciences, University of Tokyo, Bunkyo-ku, Tokyo, Japan; Bauer Research Foundation, UNITED STATES

## Abstract

Canine urothelial carcinoma (cUC) is the most common tumor of the lower urinary tract in dogs. Although chemotherapy and radical surgery have improved the overall survival, most dogs with cUC succumb to metastasis or recurrence. Therefore, the development of an effective systematic therapy is warranted. In this study, a comprehensive drug screening test using a cUC cell line was performed and the anti-tumor effect of a histone deacetylase (HDAC) inhibitor was evaluated. Comprehensive drug screening was performed on cUC cells. Based on this screening, the anti-proliferation effect of vorinostat, an HDAC inhibitor clinically applied in humans, was evaluated using several cUC cell lines in sulforhodamine B and flow cytometry assays. Western blot analysis was also performed to evaluate the degree of acetylation of histone H3 as well as the expression and phosphorylation of cell cycle-related molecules. The anti-tumor effect of vorinostat *in vivo* was evaluated using a xenograft model. Finally, immunohistochemistry was performed on acetyl-histone H3 in cUC and the relationship between the degree of acetylation and prognosis was examined using Kaplan–Meier survival analysis. Drug screening revealed that HDAC inhibitors consistently inhibited the growth of cUC cells. Vorinostat inhibited the growth of 6 cUC cell lines in a dose-dependent manner and induced G0/G1 cell cycle arrest. Western blot analysis showed that vorinostat mediated the acetylation of histone H3, the dephosphorylation of p-Rb, and the upregulation of p21 upon exposure to vorinostat. Furthermore, inhibition of tumor growth was observed in the xenograft model. In clinical cUC cases, neoplastic urothelium showed significant deacetylation of histones compared to the normal control, where lower histone acetylation levels were associated with a poor prognosis. In conclusion, the therapeutic potential of vorinostat was demonstrated in cUC. Histone deacetylation may be related to cUC tumor progression.

## Introduction

Canine urothelial carcinoma (cUC) is the most common tumor of the canine lower urinary tract. With its high invasiveness and propensity to spread to multiple regions, the mainstay for cUC treatment is systemic medication. Non-steroidal anti-inflammatory drugs (NSAIDs) and several chemotherapeutic regimens have been proposed as a first choice of treatment [1–4]. Moreover, in recent studies, radical surgery and intensity-modulated and image-guided radiation therapy have highlighted as effective locoregional control therapy [5, 6]. Although these treatments have been found to improve the overall survival, most dogs with cUC become resistant to treatment and succumb to local recurrence and/or metastasis [1–6]. Therefore, the development of an effective systematic therapy is needed.

The epigenome is a biological record of the chemical modifications of DNA and histones that do not induce changes in the DNA sequence. Representative examples of epigenetic changes include DNA methylation, histone acetylation, and chromatin remodeling [7]. These epigenetic modifications play an important role in the regulation of gene expression and cellular phenotype [7]. On the other hand, epigenetic dysregulation contributes to development and progression of cancer [7]. In humans, several studies have suggested that histone deacetylases (HDACs) are overexpressed in most tumors and that excessive HDAC activity mediates the deacetylation of histones, thereby downregulating the expression of tumor suppressor genes, such as p21^WAF1^ [8–11]. On the other hand, HDAC inhibitors have been found to have an anti-tumor effect on several tumor cell lines *in vitro* and *in vivo* in both humans and dogs [9, 10, 12–14]. As for their mechanisms, HDAC inhibitors induce the acetylation of deacetylated histones and restore the expression of tumor suppressor genes, potentially resulting in an anti-tumor effect [9, 10].

Vorinostat is a HDAC inhibitor clinically approved for treatment of human cutaneous T-cell lymphoma [15]. Recent studies and clinical trials have suggested that vorinostat has an anti-tumor effect on various hematological and solid tumors *in vitro* and *in vivo* [16–21]. Vorinostat is thought to restore the expression of several molecules related to the cell cycle (e.g. p21^WAF1^ and cyclins) and apoptosis (e.g. Bcl-2 family proteins) via histone acetylation [9–11, 22, 23].

In this study, we performed comprehensive drug screening using a cUC cell line and found that HDAC inhibitors had a strong anti-tumor effect. Subsequently, we investigated the anti-tumor mechanism of vorinostat on cUC cells *in vitro* and evaluated its anti-tumor potential using a xenograft model. Finally, we investigated the clinical implications of histone deacetylation in cUC.

## Materials and methods

### Cell culture

Three cUC cell lines, Sora, TCCUB, and Love were previously established in our laboratory and another three, NMTCC, MCTCC, and OMTCC, were kindly provided by Dr. Hoshino, Iwate University [24, 25]. The information of cell lines used in this study described in S1 Table. The cells were cultured in RPMI 1640 medium (Wako, Osaka, Japan) supplemented with 10% FBS (Life Technologies, Carlsbad, CA, USA) and the following antibiotics: 50 μg/mL gentamicin (Sigma-Aldrich, St. Louis, MO, USA) for Sora, Love, and TCCUB, or penicillin (100 unit/mL) with streptomycin (100 μg/mL) (Wako) for NMTCC, MCTCC, and OMTCC. Cells were incubated at 37°C with 5% CO_2_. Detailed description about characterization of cell lines were found in Supporting Information.

### Inhibitor screening

Drug screening was performed using SCAD inhibitor kits (kit I, ver. 3.3; kit II, ver. 2.0; kit III, ver. 1.6; kit IV, ver. 2.3) obtained from the Molecular Profiling Committee, Grant-in-Aid for Scientific Research on Innovative Areas “Platform of Advanced Animal Model Support” from the Ministry of Education, Culture, Sports, Science and Technology, Japan (KAKENHI 16H06276). Sora cells were plated at 11,000 cells/well in a 96-well plate, which was determined in a preliminary experiment to ensure logarithmic growth for 72 h. After incubation for 24 h, a total of 331 drugs were added at final concentration of 10 μM. The drugs were dissolved in DMSO at final concentration of 0.001% and same amount of DMSO was added to control well. After 48 h incubation, the cell toxicity was evaluated using a sulforhodamine B (SRB) assay [26]. Briefly, cold 10% trichloroacetic acid solution (Wako) was added to each well, and the plates were incubated at 4°C for 1 h for fixation. Then, the plates were washed four times with slow-running tap water and dried at room temperature. Subsequently, 0.057% SRB solution (Sigma-Aldrich) was added to each well. The plates were incubated at room temperature for 30 min and rinsed four times with 1% acetic acid to remove unbound dye. Tris base solution (10 mM, pH 10.5) was added to each well, and the plates were incubated for 30 min at room temperature. Absorbance at 510 nm was measured using a Bio-Rad Microplate Reader Model 550 (Bio-Rad Laboratories, Hercules, CA, USA).

### Cell growth inhibition assay

Each cUC cell line was seeded at an optimized density of 3,000–11,000 cells/well in 96-well plates before being exposed to vorinostat (Abcam, Cambridge, MA, USA) at varying concentrations (0.001–10 μM) for 48 h after an initial incubation of 24 h. The seeding conditions were determined in preliminary experiments to ensure logarithmic growth for 72 h. The drugs were dissolved in DMSO at final concentration of 0.001% and same amount of DMSO was added to control well. Cell toxicity was determined using an SRB assay. The 50% inhibitory concentration (IC_50_) was estimated with a 4-parameter logistic model using R software (ver. 3.4.0) and drc package (ver. 3.0–1) [27].

### Cell cycle analysis

Sora and TCCUB cells were exposed to 1 μM vorinostat for 24 h after an initial incubation of 24 h. Subsequently, the cells were trypsinized and fixed with 100% ice-cold ethanol for 20 min on ice. After incubation, the cells were washed with PBS and stained with 50 μg/mL propidium iodide (Sigma-Aldrich), 0.1 mg/mL RNase A (Roche Diagnostics, Basel, Switzerland), and 0.05% Triton X-100 (Sigma-Aldrich) for 40 min at 37°C. The stained cells were immediately analyzed using BD FACSverse (BD, Franklin Lakes, NJ, USA) and BD FACSuite software (BD). The proportion of each cycle phase was determined by FlowJo software.

### Western blot analysis

Western blot analysis was performed as previously described [28]. Sora and TCCUB cells were exposed to vorinostat at varying concentrations (0.1–5 μM) for 6 h or 24 h after an initial incubation of 24 h. Cells were lysed using RIPA buffer (50 mM Tris-HCl, 150 mM NaCl, 5 M EDTA, 1% Triton-X, and 0.1% sodium dodecyl sulfate [SDS]) supplemented with 10 mM NaF, 2 mM Na_3_VO_4_, and cOmplete Mini Protease Inhibitor Cocktail (Roche Diagnostics). Protein content was measured using a BCA Protein Assay Kit (Thermo Fisher Scientific, Waltham, MA, USA). Proteins (10 μg) were resolved by SDS-polyacrylamide gel electrophoresis and transferred onto polyvinylidene difluoride membranes (Bio-Rad Laboratories). The membranes were blocked in Tris-buffered saline with 0.1% Tween (TBST) containing 5% skim milk for 1 h at room temperature before incubating with the following primary antibodies at the indicated conditions in 5% skim milk (TBST-milk) or 5% bovine serum albumin (TBST-BSA): (1) polyclonal rabbit anti-Histone H3 (acetyl K9) antibody (Abcam; 1:1000, TBST-milk, 4°C, overnight); (2) polyclonal rabbit p21 antibody (Santa Cruz Biotechnology, Santa Cruz, CA, USA; 1:500, TBST-milk, 4°C, overnight); (3) monoclonal anti-phospho-retinoblastoma protein (p-Rb, Ser 807/811, Cell Signaling, Beverly, MA, USA; 1:1000, TBST-BSA, 4°C, overnight); (4) monoclonal mouse anti-actin antibody (Merck Millipore, Billerica, MA, USA; 1:10,000, TBST-milk, 4°C, overnight); (5) polyclonal anti-retinoblastoma protein (Rb, abcam, 1:500, TBST-milk, 4°C, overnight); (6) polyclonal anti-Histone H3 (abcam, 1:1000, TBST-milk, 4°C, overnight). Horseradish peroxidase-conjugated anti-mouse or anti-rabbit IgG antibody (GE Healthcare, Buckinghamshire, England; 1:10,000, TBST-milk, room temperature) was then incubated for 1 h as the secondary antibody. The membranes were developed using the ECL Prime Western Blotting Detection System (GE Healthcare) and luminescence was captured using an imaging system (ChemiDoc Image Lab, Bio-Rad Laboratories).

### Immunodeficient mouse xenograft model

BALB/ c-nu/nu mice (4-week-old, Japan SLC, Shizuoka, Japan) were maintained under specific pathogen-free conditions at 24±1°C at 40–70% humidity and a 12 h light-dark cycle throughout all the experiments. After 3 days of acclimation, mice were systemically irradiated (4 Gy) and inoculated with 6.5 × 10^6^ Sora cells. The mice were then randomly assigned to either control (n = 8) or vorinostat (150 mg/kg/day i.p., n = 9, Selleck Chemicals, Houston, TX, USA) groups. Vorinostat was dissolved in sterile distilled water containing 2% DMSO, 30% polyethylene glycol, and 5% Tween80. The same vehicle injection was performed in the control mice. All mice were treated for 28 days from the day of tumor cell injection and were humanely euthanized at day 29. Treatment regimen was determined based on previous publications [29]. Mice body weight and tumor volume were measured every 3 days. Tumor volume was calculated using the following approximation formula:

$${}^{\text{Tumor}\;\text{volume}=1/2\times \left(\text{major}\;\text{radius}\right)\times {\left(\text{minor}\;\text{radius}\right)}^{2}}$$ (1)

The study protocol was approved by the University of Tokyo Animal Care and Use Committee (approval number: P17-147). The humane endpoints were a tumor diameter 250 mm^3^, significant weight loss (15% of pre-experiment body weight), observation of clinical signs by progression of transplanted tumor cells, or drug side effects, including decreased activity, loss of appetite, shivering, and respiratory failure. The method of euthanasia used was cervical dislocation under general anesthesia induced by isoflurane. On day 29, after euthanasia, tumor masses were harvested and measured tumor volume using the following approximation formula.

Tumorvolume=(Length)×(Width)×(Height) (2)

### Immunohistochemistry

Immunohistochemistry (IHC) for acetyl-histone was performed on 4-μm thick FFPE sections of normal bladder tissues (n = 11) and cUC tissues (n = 28). Canine TCC tissues were obtained by surgical removal at the Veterinary Medical Center of the University of Tokyo (VMC-UT) between 2009–2016. Normal bladder tissue were obtained from 11 cadavers presented to the VMC-UT for routine necropsy [30]. These bladder tissue was defined as normal based on histopathological examination by Japanese domestic board certified pathologists (MT, JC and KU). Diagnosis of cUC was made based on combination of clinical findings and histopathological examination by Japanese domestic board certified veterinary pathologists (MT, JC, and KU). Slides were deparaffinized in xylene and rehydrated in gradient ethanol and water. Antigen retrieval was performed by autoclaving for 10 minutes at 121°C in 10 mM citrate buffer (pH 6.0). Endogenous peroxidase activity was blocked using 3% H_2_O_2_ in methanol at room temperature for 5 minutes. Sections were incubated with 5% skim milk in TBST at room temperature for 60 minutes. Primary antibodies were mounted onto slides for 10 minutes at room temperature. The antibody used was polyclonal rabbit anti-histone H3 (acetyl K9) antibody (Abcam, 1:500). Slides were washed with TBST and then incubated with EnVision polymer reagent for rabbit (Dako) at room temperature for 60 minutes. Immunohistochemical reactions were visualized using DAB (Dako) and nuclei were counterstained with hematoxylin. The positive cells were counted in five independent high-power (×400) fields (HPF). To identify positive cells, the threshold of staining intensity was set by Image J software and the percentage of positive cells was calculated by dividing by the total number of normal epithelial cells or tumor cells in each field (1–100%). Slides without primary antibody served as the negative control, and normal bladder tissue itself was considered positive control because cells with lineage specification should have epigenetic modifications including histone acetylation to some extent as long as cells are not transformed or reprogrammed [31].

### Survival analysis

The clinical features and outcomes of 28 cUC cases were obtained from their medical records or from fax interviews with the corresponding veterinarians (S2 Table). Cases of perioperative death were not included. Overall survival (OS) was defined as the period from surgery until death. Progression free survival (PFS) was defined as the time from surgery to recurrence/metastasis or death at the end of the study. The cases were categorized into either a high acetylation group and a low acetylation group according to the percentages of positive cells (cut-off value = 50%). The cut-off value was determined using the online tool Cut-off Finder [32].

### Statistical analysis

All data are shown as the mean ± standard deviation (SD). To compare difference between two groups, Two-sided Student’s t-test and ANOVA test were performed. Fisher’s exact test was performed to evaluate the enrichment significance of the results from the inhibitor screening. Sample size calculation was done for xenograft study with a power of 0.8 and level of significance of 5% (p<0.05) based on the results from the previous publication [33]. Chi square tests were performed to compare clinical parameters between low and high histone H3K9 acetylation groups. All analysis above was done using R software (https://www.R-project.org/). In addition, Kaplan–Meier analysis and long-rank test were performed using R software and the cmprsk R package (ver. 2.2–7) [34]. P ≤ 0.05 was considered statistically significant.

## Results

### Characterization of cUC cell lines

The cell lines were evaluated for cUC specific BRAF^V595E^ mutation [35] and expression of cUC marker molecules (UPK3A, UPK3B and KRT7) [36]. Five out of Six cell lines, except for OMTCC, carry BRAF^V595E^ mutation (S1 Fig). All cells showed strong expression of at least two cUC markers when compared to normal peripheral blood mononuclear cells and a canine melanoma cell line which are used as normal and neoplastic negative controls (S2 Fig). Information of dogs of origin of the cell lines and doubling times were summarized in S1 Table together with the results above. The cell lines were also negative for a mycoplasma test.

### Comprehensive drug screening against cUC cell lines

**Fig 1:**
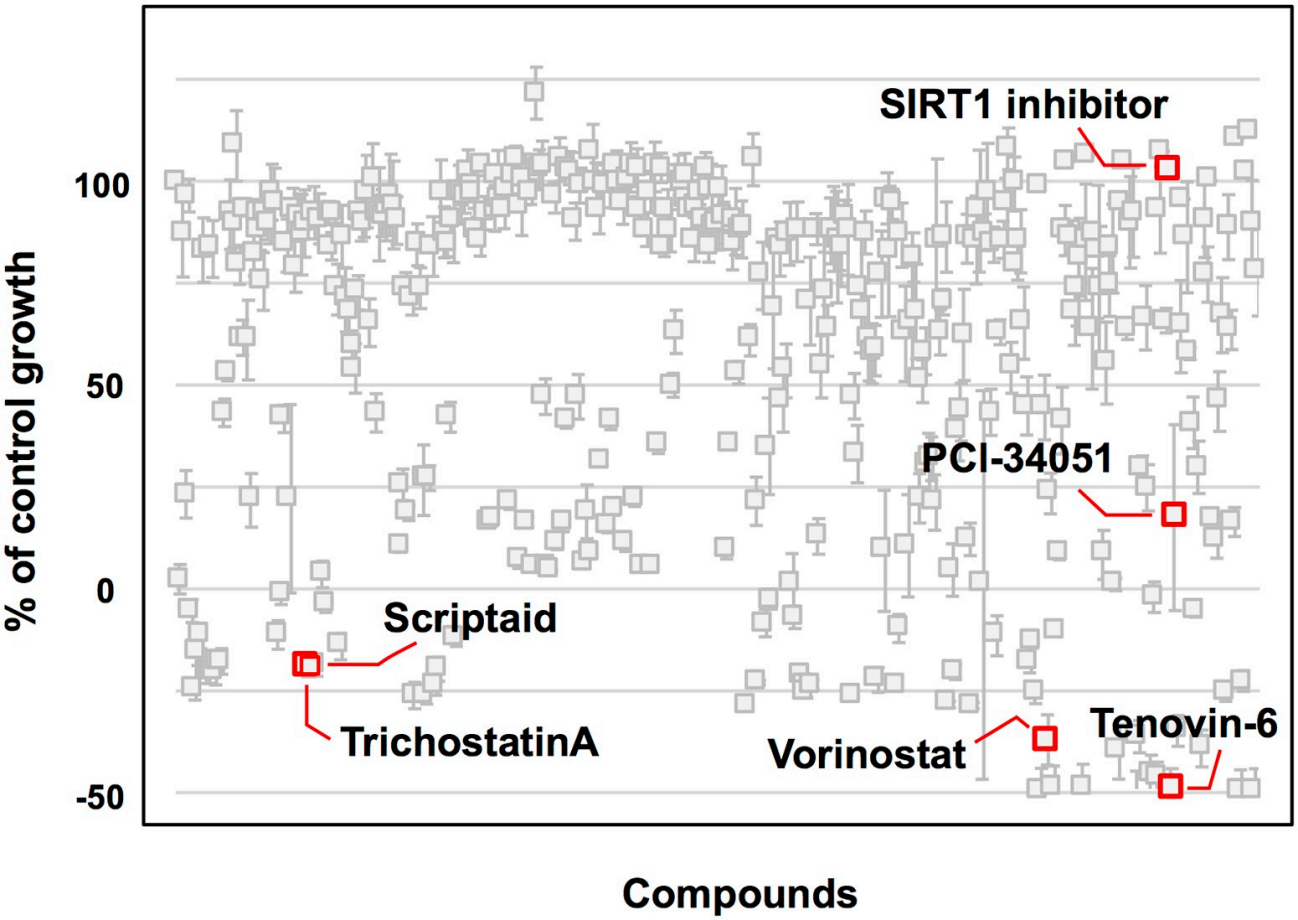
The result of drug screening (SCAD inhibitor kits I-IV). The y-axis represents cell survival (%). Each point represents the result for each compound. HDAC inhibitors are shown in red and labeled with the name of the compounds. The data consists of 4 technical replicates and the values are shown as the mean value ± standard error of the mean.

To identify novel drugs for use against cUC cells, comprehensive drug screening was performed using SCADs inhibitor kits Ⅰ-Ⅳ (Fig 1). Sora cell line was used for this screening. Of the 331 drugs tested, 51 inhibited cell growth in the Sora cell line by over 90% at 10 μM. HDAC inhibitors were found to be significantly enriched in these agents (4/51 vs. 2/280; P < 0.01). The target molecules and cell survival of the HDAC inhibitors included in the screening kits are shown in Table 1. Vorinostat, a clinically approved HDAC inhibitor used in humans, was selected for subsequent experiments.

**Table 1:** HDAC inhibitors in the SCAD inhibitor kits, their target HDACs, and anti-proliferative effect against Sora cells (%).

Drug name	Target HDACs	Cell survival (%)
Vorinostat	HDAC 1, 2, 3, 6	6.5
Trichostatin A	HDAC 1, 2, 3, 4, 6, 10	6.2
Scriptaid	HDAC 1, 3, 8	6.2
PCl-34051	HDAC 8	40.9
SIRT-1 inhibitor	SIRT-1	98.7
Tenovin-6	SIRT-1, 2, 3	0

### Vorinostat inhibited cUC cell growth and induced G0/G1 cell cycle arrest

**Fig 2:**
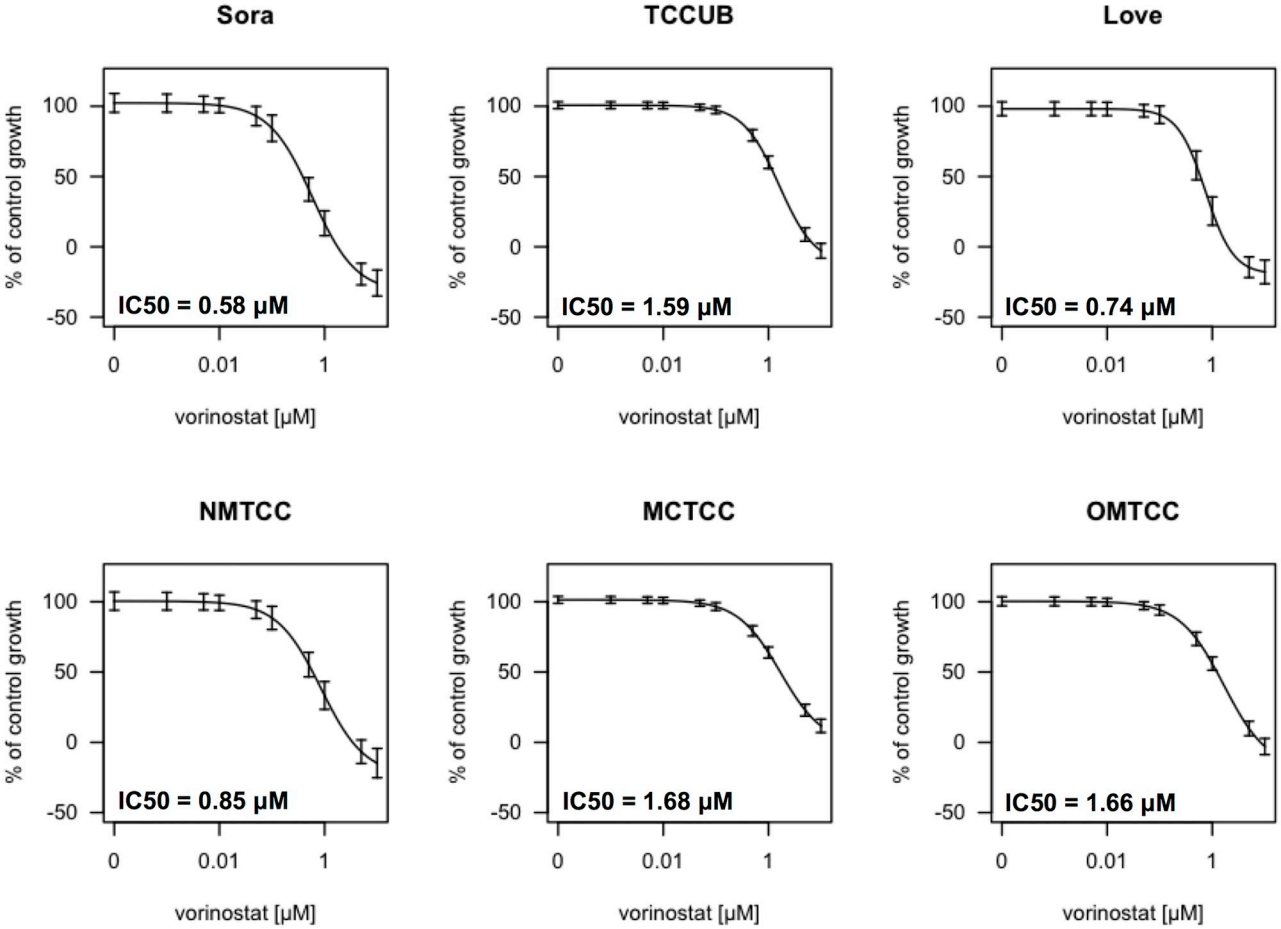
Growth inhibition of vorinostat on six cUC cells. The dose-response curves of vorinostat for six cUC cell lines. The data consists of 8 technical replicates and the experiments were repeated independently three times. The values are shown as the mean value ± standard error of the mean.

**Fig 3:**
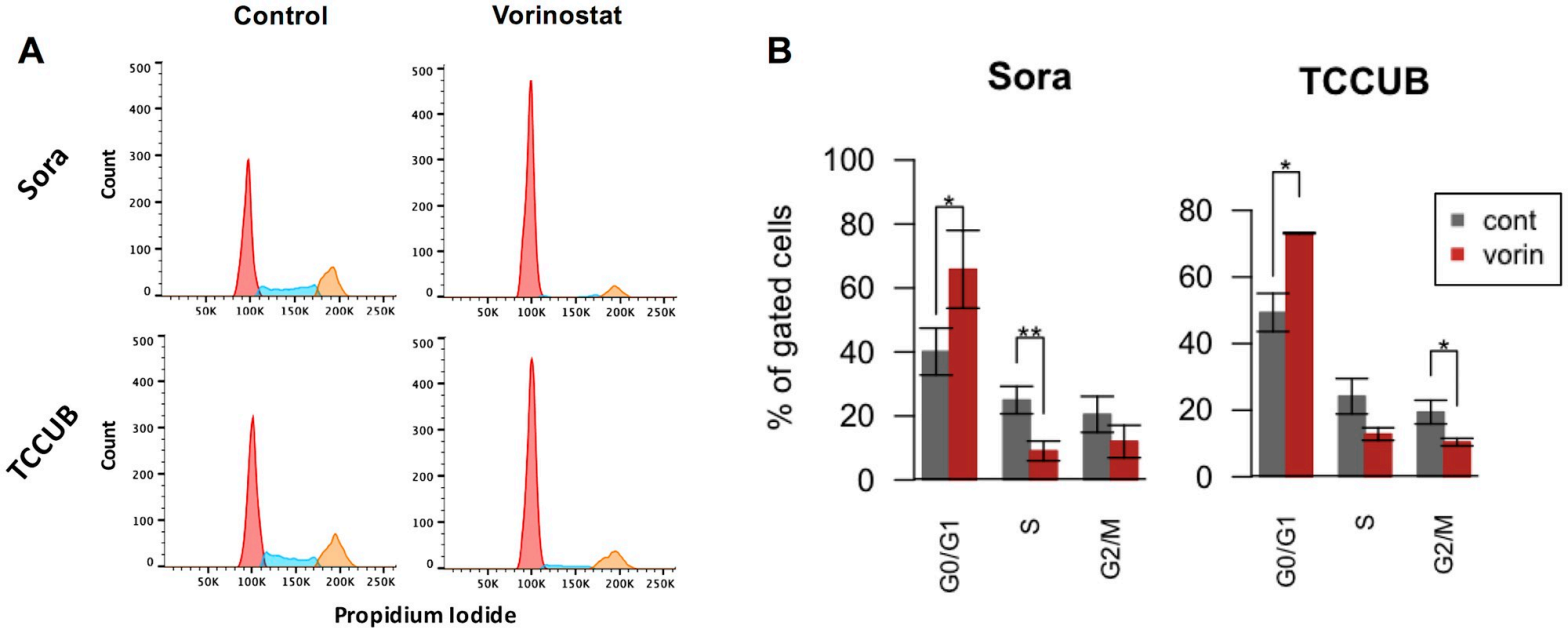
Effect of 24 h vorinostat exposure on cell cycle regulation. A: Representative result of flow cytometric cell cycle analysis using propidium iodide. The x-axis indicates PE intensity (propidium iodide). After data requisition and pre-process by FACSuite, the proportion of each cycle phase was determined by FlowJo software. Red: G0/G1 phase, light blue: S phase, yellow: G2/M phase. B: Summary of the effect of vorinostat treatment on the cell cycle (*P < 0.05, **P < 0.01, t-test, control vs vorinostat).

Vorinostat inhibited cell growth in six cUC cell lines in a dose-dependent manner (Fig 2). The IC_50_ values for Sora, Love, TCCUB, OMTCC, NMTCC, and MCTCC were 0.57, 1.57, 0.21, 0.86, 1.69, and 1.57 μM, respectively, after 48 h of exposure. Next, we investigated the effect of vorinostat on the cell cycle of cUC cell lines using flow cytometric analysis (Fig 3A). Sora and TCCUB cells were used as representatives of one with IC50 value < 1 uM and one with IC50 value > 1 uM, respectively. Love, the most sensitive cell line to vorinostat, had apparent aneuploidity, which is not suitable for cell cycle analysis. The number of G0/G1 population cells was significantly increased and S and G2/M population cells were significantly decreased by vorinostat in these cells (Fig 3B).

### Vorinostat induced histone acetylation and the expression of cell-cycle related molecules

**Fig 4:**
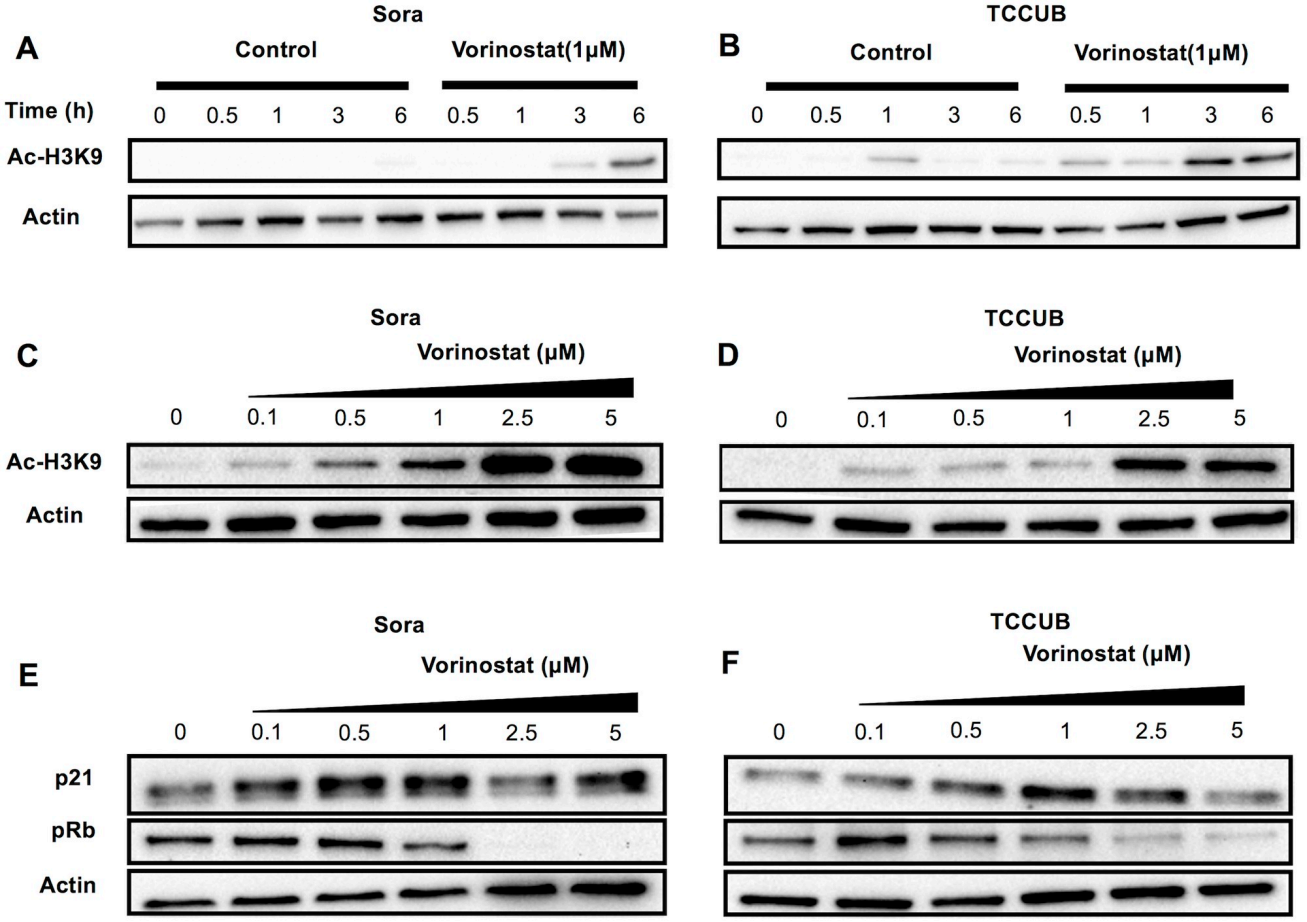
Representative results of western blot analysis of H3 acetylation (A-D) and cell cycle-related molecules, p21 and p-Rb (E, F). Cells were incubated with 1 μM vorinostat for 0.5–6 h (A, B), with 0.1–5 μM vorinostat for 24 h (C, D), and with 1 μM vorinostat for 24 h (E,F). Whole cell lysates were analyzed by western blot analysis.

The anti-tumor effect of vorinostat is considered to depend on the induction of histone acetylation. Therefore, we examined the changes in the histone acetylation levels of cUC cells after vorinostat exposure. Sora and TCCUB cells were used as in cell cycle analysis. Vorinostat induced histone H3K9 acetylation in a time-dependent (Fig 4A and 4B) and dose-dependent manner in Sora and TCCUB cells (Fig 4C and 4D). In previous studies, vorinostat induced G0/G1 cell cycle arrest by the upregulation of p21 ^WAF1^ and dephosphorylation of Rb is related to the shifts from G1 to S phase [13,14]. Therefore, we investigated the changes in the expression of p21 ^WAF1^ and phospho-Rb after vorinostat exposure. Increased p21^WAF1^ expression levels and the dephosphorization of Rb were observed as a result of vorinostat exposure (Fig 4E and 4F). These changes in Acetylated H3K9 and phosphorylated Rb were not due to decrease in total H3 and total Rb expression, respectively (S3 Fig).

### Vorinostat inhibited cUC tumor growth in a xenograft mouse model

**Fig 5:**
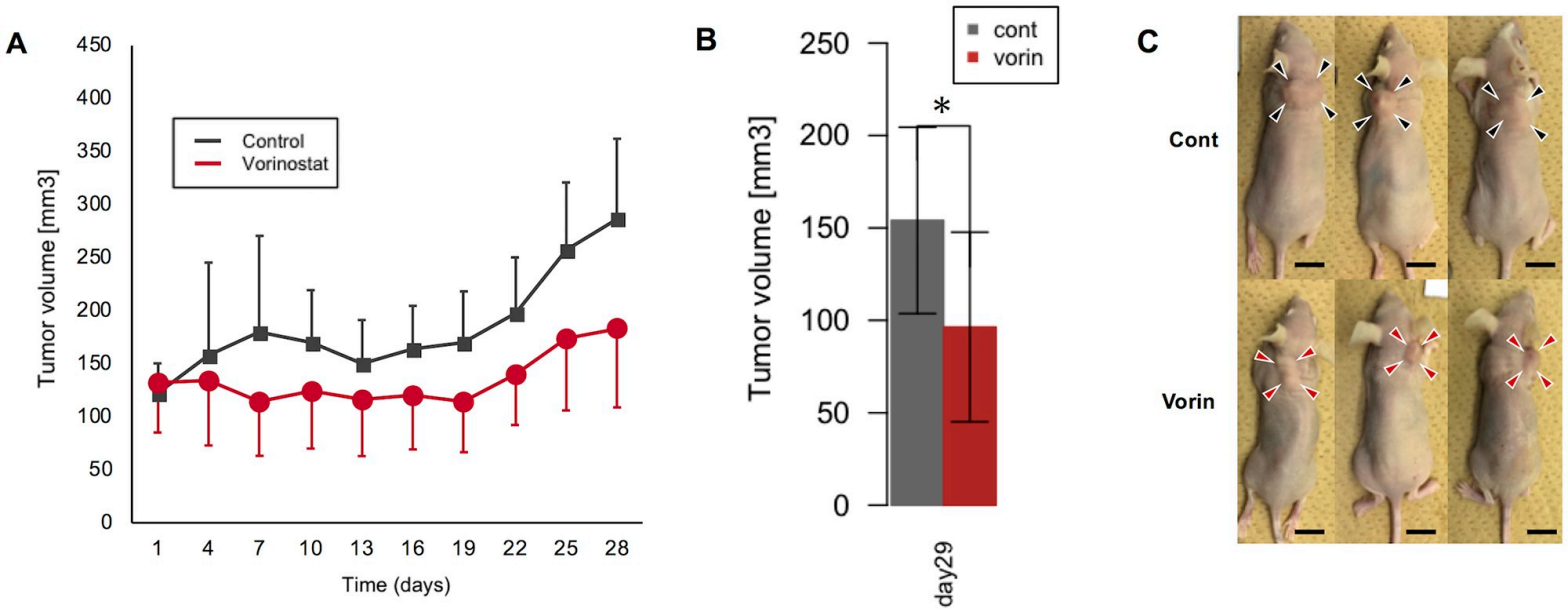
The effect of vorinostat in cUC xenograft model. Changes in tumor volume (A, p = NS by ANOVA, control vs. vorinostat), tumor volume after harvested on day 29 (B, *p < 0.05 by student-t test, control vs. vorinostat) and the representative macroscopic appearance of xenografted mice on day 29. (C, scale bar = 1 cm).

To further investigate the anti-tumor effect of vorinostat, a xenograft study was performed. Sora cell line was chosen based on its sensitivity to vorinostat with information about mechanism of action described above. The result showed that vorinostat significantly inhibited the tumor growth after 28 days of treatment (Fig 5A and 5B). As for adverse effect, the weight of the vorinostat group was significantly lower than the control group on day 13 (S4 Fig), and two mice died in the vorinostat group without presenting any adverse symptoms.

### H3K9 deacetylation was observed in cUC tissue in association with shorter PFS

**Fig 6:**
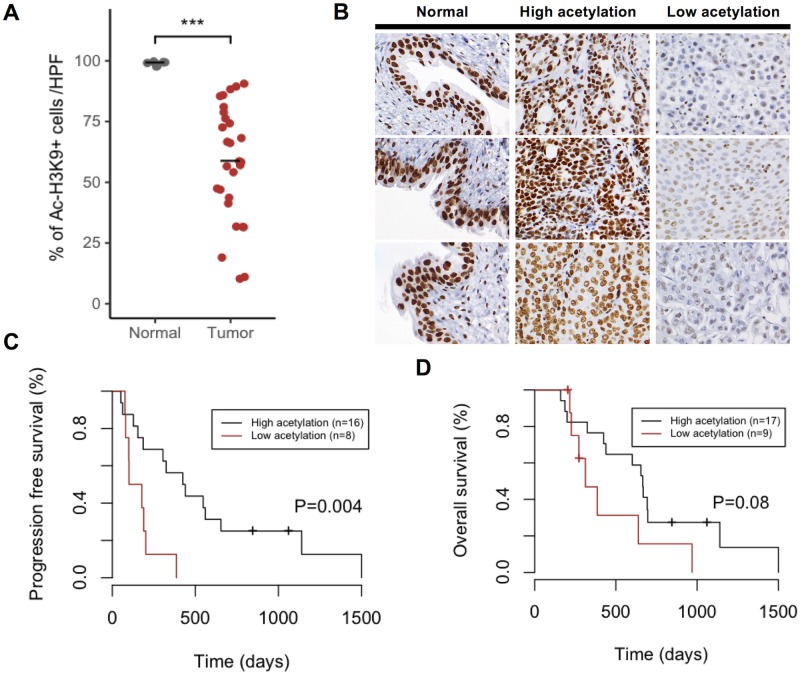
Immunohistochemistry of cUC tissues and correlation between percentages of acetylated cells and prognosis. A: The percentage of acetyl-histone H3K9 positive cells in normal bladder tissues and cUC tissues (*** P < 0.001, Mann–Whitney U test, normal bladder tissues vs cUC tissues). B: Representative images of immunohistochemical staining of acetyl-histone H3K9 in normal bladder tissues and cUC tissues (high acetylation group and low acetylation group). C: Kaplan–Meier survival analysis of PFS (C) and OS (D) in cUC cases (log-rank test, high acetylation group vs low acetylation group). Two cases (OS) and four cases (PFS) were excluded because of perioperative death and due to the unavailability of data.

Although the normal bladder epithelium showed intense H3K9 acetylation, various degrees of H3K9 deacetylation were observed in cUC tissues (Fig 6A). The representative immunohistochemical results are shown in Fig 6B. Next, we investigated the extent of H3K9 deacetylation in relation to cUC prognosis. Information of cases can be found in S2 Table. cUC cases were classified either as high acetylation (>50%) or low acetylation (<50%). The degree of H3K9 deacetylation was significantly correlated to shorter PFS (Fig 6C). This tendency was also observed for OS, although it was not statistically significant (Fig 6D). Between high and low histone H3K9 groups, difference in other features such as TNM stage, tumor location, type of surgery, and BRAF mutation status was not found.

## Discussion

In this study, drug screening using cUC cell lines was used to demonstrate the potential of HDAC inhibitors as a novel molecular drug for the treatment of cUC. Vorinostat was found to inhibit the cell growth of cUC cell lines both *in vitro* and *in vivo*. Moreover, exposure to vorinostat restored histone acetylation in cUC cells. Simultaneously, vorinostat increased the expression of p21 and induced the dephosphorylation of p-Rb with cell cycle arrest, suggesting that this was one of the molecular mechanism of the anti-tumor effect of the drug. Furthermore, histone deacetylation was aberrantly observed in cUC tissues, and lower histone acetylation levels were related to a poor prognosis.

Epigenetic dysregulation, including histone deacetylation, has been recently associated with tumor progression [7]. Although several studies have reported that human UC has epigenetic alterations and the therapeutic potential of HDAC inhibitors, epigenetic dysregulation and the efficiency of HDAC inhibitors on cUC have not yet been documented [37–40]. In this study, cUC cell lines showed marked sensitivity to HDAC inhibitors and histone deacetylation was ubiquitously observed in cUC tissues, suggesting that epigenetic dysregulation may play an important role in cUC progression. The accumulation of epigenetic mutations, such as the overexpression of HDAC, may occur in cUC cells and tissues.

HDACs include HDAC1-11 and SIRT1-7, and they are classified into three different classes: HDAC 1, 2, and 3 [9]. Scriptaid, trichostatin A, vorinostat, and tenovin-6 inhibit multiple HDACs and were found to suppress over 90% of cell growth in Sora cells. However, PCl-34051 and SIRT-1, which inhibit specific HDACs, were not found to strongly suppress cell growth. The overexpression of HDACs has been previously observed in tumors, where the expression patterns vary according to different tumor types and patients [8]. Although we did not determine the expression levels of each HDAC subtype in the cUC cells, the difference in sensitivity between HDAC inhibitors may be related to the differences in expression levels. Further study is needed in order to determine the relationship between the expression of each HDAC subtype and sensitivity to HDAC inhibitors, coupled with genetic engineering such as RNAi, to elucidate more specific targets in cUC cells.

In humans, vorinostat has been shown to inhibit cell growth of various hematological and solid tumors *in vitro*, including lymphoma, myeloma, leukemia, colon and pancreas cancer, non-small cell lung carcinoma, and bladder cancer, with IC_50_ values ranging from approximately 0.1 to 10 μM [41–44]. In this study, the median IC_50_ of vorinostat for cUC cell lines was 1.18 μM (0.58–1.68 μM), a value comparable to that reported in the human blood peak concentration (1–2 μM). Although the *in vitro* experiments in this study did not completely reproduce pharmacokinetics of vorinostat *in vivo* considering its short half-life in human blood, it was shown that vorinostat could inhibited tumor growth *in vivo*. These results suggested clinical potential of the drug against canine cUC.

Vorinostat has several mechanisms for its anti-tumor effects, such as cell cycle arrest or apoptosis, which vary according to tumor type [9, 11, 22, 23]. In this study, vorinostat induced G0/G1 cell cycle arrest in cUC cells, and western blot analysis revealed that vorinostat increased the expression of p21^WAF1^ and induced the dephosphorization of Rb. Vorinostat-mediated increases in p21^WAF1^ expression have been found in several human cancers and plays a critical role in G0/G1 cell cycle arrest [9–11]. Rb also controls cell cycle progression from the G1 to the S phase and the dephosphorization of Rb via the upregulation of p21, which induces G0/G1 cell cycle arrest [45, 46]. This suggests that vorinostat induces p21^WAF1^ gene expression and Rb dephosphorization via the restoration of histone acetylation in cUC cells. On the other hand, given that the sub-G1 population was not influenced by vorinostat exposure, vorinostat may not induce apoptosis in cUC cells. These data suggest that the major anti-tumor mechanism of vorinostat in cUC cells is the induction of G0/G1 cell cycle arrest.

Vorinostat also significantly inhibited cUC growth *in vivo*. Although adverse effects were observed in this study, a possible explanation for this a comparatively long-term administration compared to other studies [29, 47]. In the safety assessment of vorinostat for use in dogs, no adverse effects were observed up to the 6-fold maximum recommended dose in human [48]. Therefore, vorinostat is safe for use as an effective therapy against cUC.

Previous studies have suggested that the hypoacetylation of histones is related to a poor prognosis in human acute lymphoblastic leukemia, cervical carcinoma, and renal cell carcinoma [49–51]. Therefore, we investigated whether the level of acetyl-histone H3K9 affects prognosis. The Kaplan–Meier estimation method and log-lank test found that lower levels of histone acetylation in tumor cells were correlated with shorter PFS (Fig 6C), suggesting that the deacetylation of histones is related to a poor prognosis. Although this tendency was also observed for OS, there was no significant difference (Fig 6D). This could be attributed to a small sample size. Moreover, we only investigated acetyl-histone H3K9. As such, further studies with large sample sizes will be required to elucidate the relationship between histone modifications of other acetylated sites (e.g. H3K18, H3K27, and H4) and prognosis.

A recent study found that the BRAF^V595E^ mutation, which is homologous to human BRAF^V600E^, was detected in ~80% of cUC cases [35]. In humans, BRAF^V600E^ mutant melanoma cells have been reported to be resistant to HDAC inhibitors [52, 53]. Although five out of six cell lines used in this study carry BRAF^V595E^ mutation, mutant cell lines showed sensitivity to vorinostat, similarly to the BRAF wild type cell line (OMTCC). Furthermore, no differences were found regarding histone acetylation levels between BRAF^V595E^ and wild-type tumors in cUC tissues. These results indicate that the molecular mechanisms of cUC may differ from human melanoma. As such, further study is required to elucidate the relationship between BRAF mutations and epigenetic histone acetylation.

## Conclusions

In this study, drug screening and histone acetylation analysis of cUC tissues were used to demonstrate that histone deacetylation may be associated with cUC tumor progression. We also demonstrated that vorinostat has an anti-tumor effect on cUC cells both *in vitro* and *in vivo*. Further investigation will be required in order to elucidate the detailed anti-tumor mechanisms of this treatment and its effectiveness in clinical studies.

## Supporting information

S1 Fig Digital PCR-based genotyping of BRAF mutation in cUC cell lines. X-axis: VIC, wildtype BRAF gene. Y-axis: FAM, BRAF^V595E^. Presence of blue and green clusters indicates that a cell line carries BRAF mutation.
(TIFF)

S2 Fig Expression of cUC markers in cUC cell lines and other cells. PBMC, peripheral blood mononuclear cells. KMEC, a canine melanoma cell line.
(TIFF)

S3 Fig Western blot analysis of Ac-H3K9, total-H3, p-Rb, and total-Rb in Sora cell line after vorinostat exposure. Cells were treated with vorinostat for 24 h at 0.1, 0.5, 1.0, 2.5, 5 μM. Actin was used as a internal control.
(TIFF)

S4 Fig Changes in body weight in xenograft mice model. **P < 0.01 (t-test, Cont vs. Vorin).
(TIFF)

S1 Table The information of cell lines used in this study. (XLSX)

S2 Table The information of cUC patients. (XLSX)

S1 File Supplementary materials and methods. (DOCX)
