# Supplementary figures and images for: Anti-tumor effects of the histone deacetylase inhibitor vorinostat on canine urothelial carcinoma cells
Source: PLoS One. 2019 Jun 17;14(6):e0218382. doi: 10.1371/journal.pone.0218382 (PMC6576781; doi:10.1371/journal.pone.0218382)

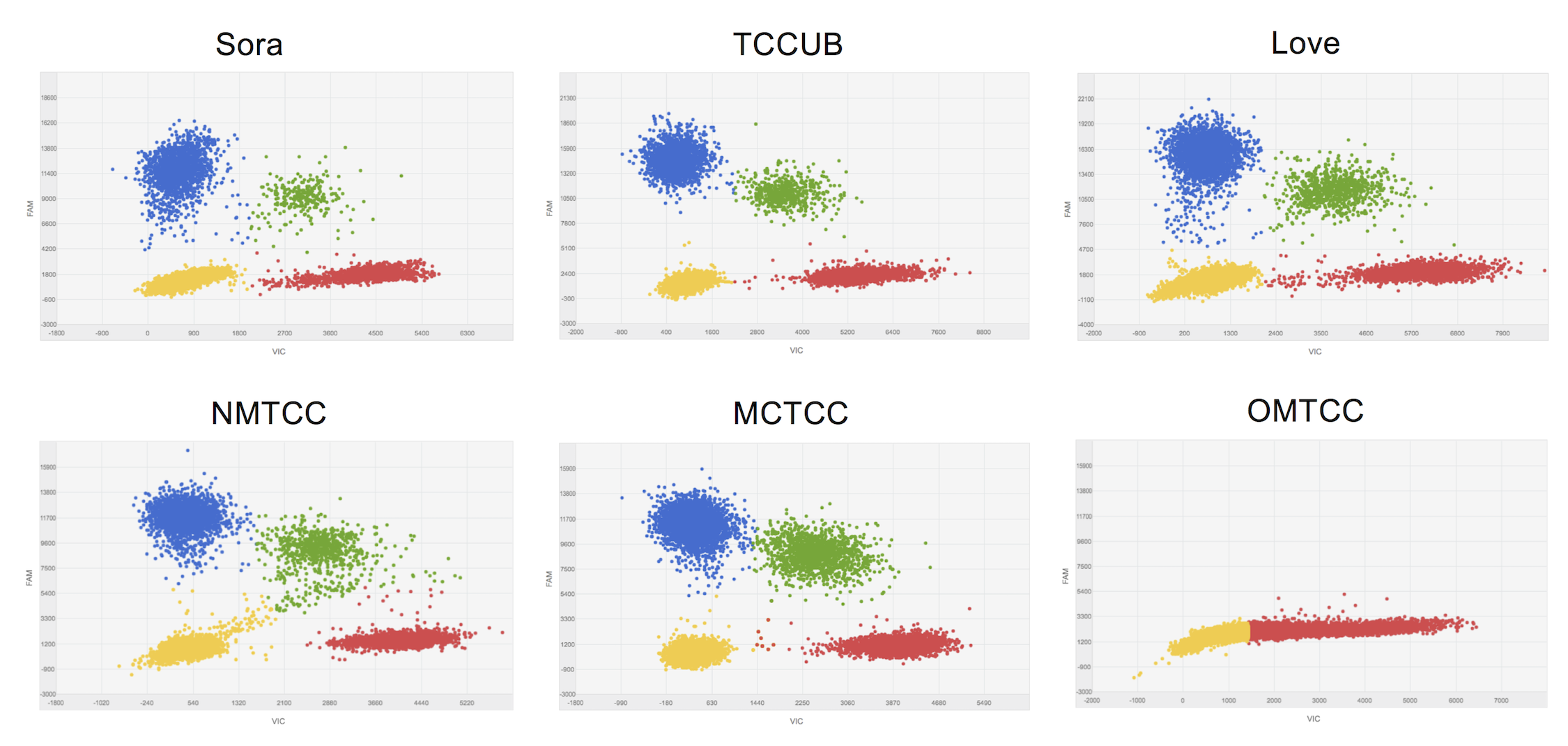

Supplement: S1 Fig — X-axis: VIC, wildtype BRAF gene. Y-axis: FAM, BRAFV595E. Presence of blue and green clusters indicates that a cell line carries BRAF mutation. (TIFF) [file pone.0218382.s001.tiff]

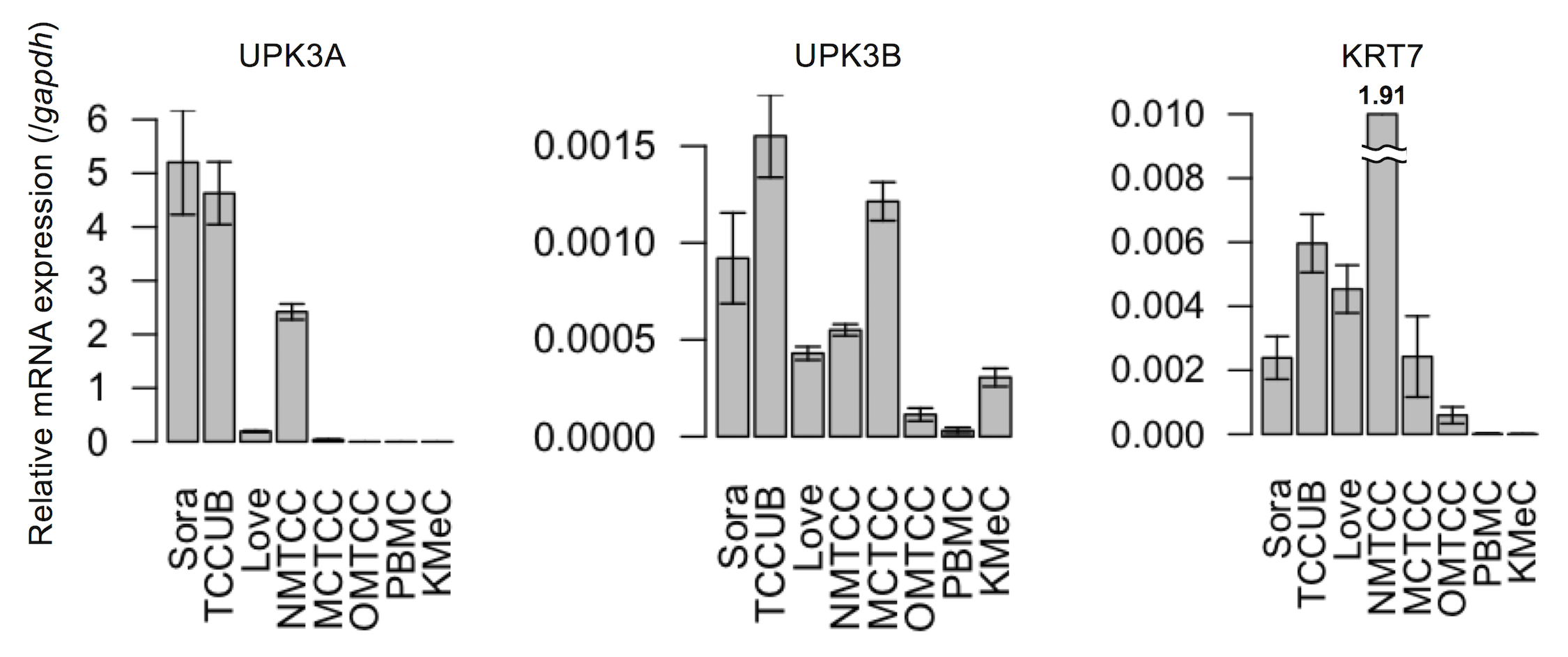

Supplement: S2 Fig — PBMC, peripheral blood mononuclear cells. KMEC, a canine melanoma cell line. (TIFF) [file pone.0218382.s002.tiff]

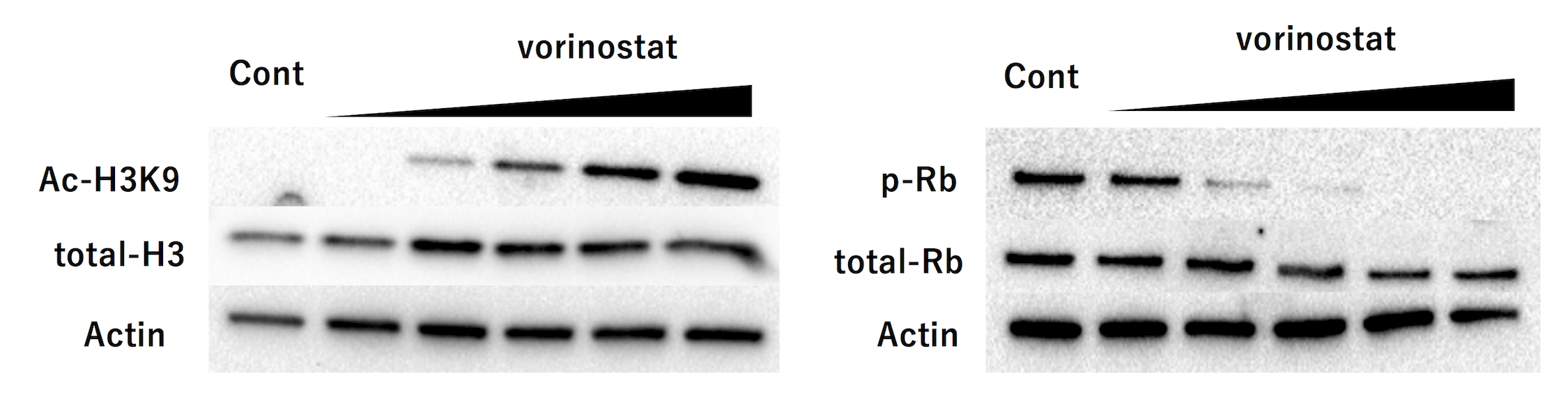

Supplement: S3 Fig — Cells were treated with vorinostat for 24 h at 0.1, 0.5, 1.0, 2.5, 5 μM. Actin was used as a internal control. (TIFF) [file pone.0218382.s003.tiff]

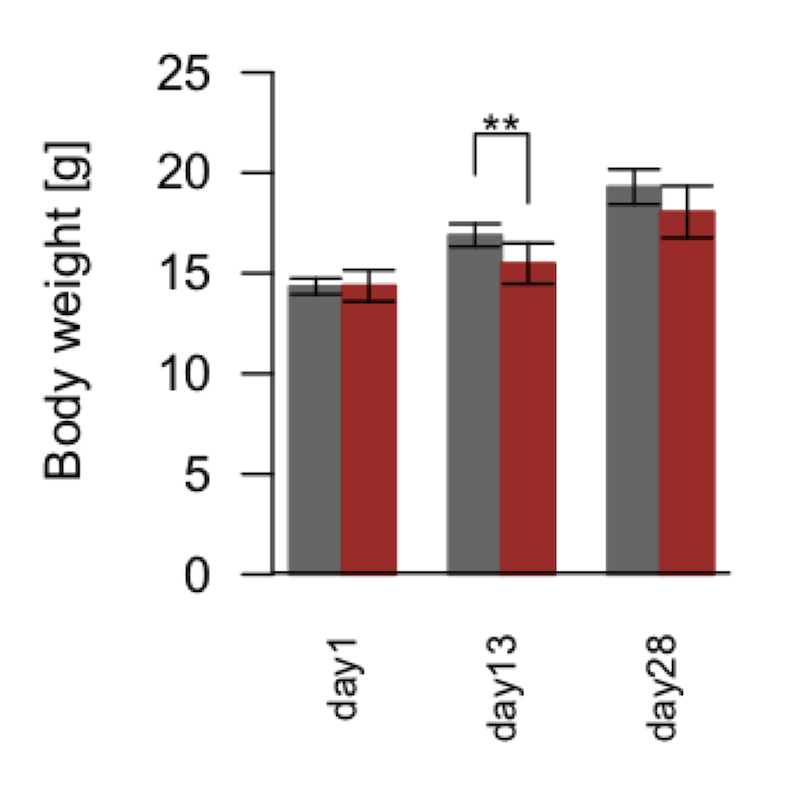

Supplement: S4 Fig — **P < 0.01 (t-test, Cont vs. Vorin). (TIFF) [file pone.0218382.s004.tiff]
