# Supplementary material for: Anti-tumor effects of the histone deacetylase inhibitor vorinostat on canine urothelial carcinoma cells
Source: PLoS One. 2019 Jun 17;14(6):e0218382. doi: 10.1371/journal.pone.0218382 (PMC6576781; doi:10.1371/journal.pone.0218382)
Supplement: S1 File — (DOCX) [file pone.0218382.s007.docx]

**SUPPLEMENTARY MATERIALS AND METHODS**

**Detection of BRAF^V595E^ mutation of cUC cell lines**

Genomic DNA was extracted from cells using the DNeasy Blood & Tissue (QIAGEN, Hilden, Germany) according to the manufacturer’s protocol. Digital PCR was performed using 2x Quantstudio3D Mastermix, 40x Assay Mix containing probes and primers for the detection of both BRAFV595E and the wild-type gene, and ProFlex Base (Thermo Fisher Scientific, Waltham, MA, USA). Probe and primer sequence were as follows; WT_VIC (5’-TAGCCACAGTGAAATC-3’), MT_FAM(5’-CCACAGAGAAATC-3’), BRAF forward (5’-CATGAAGACCTCACAGTAAAAATAGGTGAT-3’), and BRAF reverse (5’-TGGGACCCACTCCATCGA-3’). PCR condition was as follows: 10 min at 96 °C, 39 cycles of 60 °C for 2 min and 98 °C for 30 sec, and 2 min at 60 °C. Raw data was obtained and analyzed using the Quantstudio 3D Digital PCR system (Thermo Fisher Scientific) and the Quantstudio3D Analysis Suite software (Thermo Fisher Scientific).

**Quantification of mRNA expression of cUC specific markers**

Total RNA was extracted from cells in exponential growth phase using a RNA extraction reagent (TRI Reagent, Cosmo Bio, Tokyo, Japan). Subsequently, complementary DNA (cDNA) was synthesized using a reverse transcriptase (ReverTra Ace, Toyobo, Osaka, Japan). Real-time PCR was performed using a premix reagent (THUNDERBIRD SYBR qPCR Mix, Toyobo), specific primers (Thermo Fisher Scientific), and a Real-time PCR system (StepOnePlus, Thermo Fisher Scientific). Primers were used to detect the expression of Uroplakin 3A (UPK3A), Uroplakin3B (UPK3B) and Keratin7 (KRT7). The primer sequences were as follows; (a) UPK3A forward (5’-ACGGAGTACAGGTTCAAGTACG-3’) and reverse (5’- TCGTTTCCTGGTCAGAGCTG-3’); (b) UPK3B forward (5’- AGTTTAGCACCACCTTCAGC-3’) and reverse (5’- AGCTGCCTTGATGTTGACAC-3’); (c) KRT7 forward (5’- AGGAGCATTCGGAAATGAGC-3’) and reverse (5’- AAGTCTTGGTATCAGGGATGGG-3’). Thermocycling was performed with 40 cycles of denaturation at 95ºC for 15 s and extension at 60ºC for 60 s. Relative expression values were determined following normalization to *gapdh* expression, which served as an internal control. The expression of these markers was concluded in comparison with archived RNA from canine peripheral blood mononuclear cells and a canine melanoma cell line (KMeC) as negative controls.

**Doubling time**

Cells were plated in 6 well plate at a concentration of 1×10^4^ cells/cm^2^ and the number of viable cells was determined every 24 h under microscope using trypan-blue staining. The doubling time was calculated in the exponential growth phase.

**Mycoplasma detection**

MycoAlert mycoplasma detection kit (Lonza Bioscience, Basel, Switzerland) was used according to manufacturer’s instruction.
